# The Effect of Psychoeducation on Anxiety in Subsequent Pregnancy Following Stillbirth: A Quasi-Experimental Study 

**Published:** 2018-03

**Authors:** Mehrnegar Azogh, Mansour Shakiba, Ali Navidian

**Affiliations:** 1 Department of Midwifery, School of Nursing and Midwifery, Zabol University of Medical Sciences, Zabol, Iran; 2 Department of Psychiatry, School of Medicine, Zahedan University of Medical Sciences, Zahedan, Iran; 3 Pregnancy Health Research Center, School of Nursing and Midwifery, Zahedan University of Medical Sciences, Zahedan, Iran

**Keywords:** Pregnancy Subsequent to Stillbirth, Pregnancy Anxiety, Psychoeducation* Women

## Abstract

**Objective:** We aimed to determine the effect of psychoeducation on women’s anxiety in subsequent pregnancy following stillbirth.

**Materials and methods:** This two-arm, semi-experimental study was conducted on 100 women with subsequent pregnancy after stillbirth who visited the healthcare centers affiliated to a university of medical sciences in southeast of Iran in 2017. The eligible women were selected by using the convenience sampling method and were randomly divided into the intervention and control groups. The intervention group attended four psychoeducation sessions during four weeks according to the determined content. On the other hand, the control group received the routine care education. After eight weeks, data were collected using Pregnancy Related Anxiety Questionnaire (PRAQ). To analyze the data, independent t-test, Paired t-test and Chi-square U test, were run in SPSS, version 21.

**Results:** No significant differences were observed between the study groups in terms of demographic characteristics (p > 0.05). Although the mean score of anxiety was not significantly different in the intervention and control groups prior to the psychoeducation sessions (p = 0.83), it was significantly lower in the intervention group after the psychoeducation intervention, compared to the control group (50.64 ± 20.05 vs. 63.54 ± 22.90; p = 0.0001).

**Conclusion:** Psychoeducation intervention could diminish anxiety in women with subsequent pregnancy after stillbirth. Therefore, we recommend incorporating the components of psychoeducation related to the special needs of this group of women as a part of the routine prenatal care and educating healthcare providers to use these interventions.

## Introduction

Stillbirth is a sad story affecting almost 1% of pregnancies. In fact, one of the most devastating losses is losing a baby, even if the child is still inside the mother’s womb. This loss could have severe adverse effects on the family, especially the mother (1).

Despite the advancements in maternity care, the rate of stillbirth has not significantly decreased across different countries such as England, compared to the past two or three decades (2). Reports on stillbirth are limited, and 98% of the cases of stillbirth occur in low- and middle-income countries, and 67% of them occur in rural families (3). In Iran, the rate of stillbirth is about 11.7-27 in 1000 cases in various cities (4).

Understanding about maternal grief following stillbirth is inadequate, and people, especially maternal support systems, believe that after a short while, the problem would be resolved and the death process ends. Therefore, pregnancy subsequent to stillbirth is emphasized, and some hold that pregnancy after pregnancy loss could dwindle the mother’s grief (5). Accordingly, their highest aspiration is to have another child to help move on from the loss of the previous child (6). 

The interval between pregnancy and stillbirth is highly different, ranging between 6 weeks and 18 years. In general, 59-86% of women want to get pregnant after loss (7). Some of the characteristics of pregnancy after pregnancy loss are elevated maternal anxiety and emotional vulnerability. In addition, psychological distress is recognized as one of the general health concerns in such pregnancies and is associated with a wide range of adverse outcomes including premature birth and low birth weight (8, 9).

While pregnancy is an opportunity for experiencing positive emotions and promising expectations for having a new child, pregnancies subsequent to stillbirth are the opposite and it is not considered equal as having a child. In these cases, mothers hope for a positive outcome and fear a negative outcome in tandem (10). Women with pregnancy subsequent to stillbirth have less confidence in their pregnancy, compared to normal women, and are extremely concerned about whether their child will live or not and their self-perception changes; they might even experience post-traumatic stress disorder during the new pregnancy (11).

In these cases, the parents disguise the pregnancy and postpone informing others (10). These mothers are more concerned about the health of their infant, and they cannot distinguish the new child from the previous stillbirth (11). Increased anxiety in these mothers leads to their frequent and unnecessary physician and healthcare center visits for tests and ultrasound evaluation to ensure the health of the child, imposing additional costs on the family and healthcare systems (2). 

Management of pregnancies subsequent to stillbirth is often different and significantly more difficult, compared to primiparous women (5). Therefore, gynecologists of the Royal College of the United Kingdom proposed that subsequent pregnancy following stillbirth must be regarded and managed as a high-risk pregnancy (2). An international study conducted across 40 middle- and high-income countries in 2016 indicated that only 10% of the women received psychological counseling in subsequent pregnancies after stillbirth and miscarriage (12). Despite all the recommendations, the lack of a defined healthcare trajectory for such women is felt all over the world (6). In some countries, such as Australia, specific clinics for pregnancy subsequent to stillbirth and neonatal have been established, which has significantly helped these women (13). Effectiveness of various methods, such as mindfulness, cognitive-behavioral therapy, psychoeducation, and stress management, in reducing the negative psychological outcomes of mothers (e.g., reduced anxiety and increased maternal-fetal bond in normal pregnancies, especially in primiparous women) has been confirmed in various studies in Iran and other countries such as Karamouzian et al. (2013), Khanzadeh et al. (2016), Thoma et al. (2014), and Fenwick et al. 2015 (14-17).

Nonetheless, these psychological interventional methods have less been evaluated in pregnant women with a history of stillbirth or miscarriage. Meanwhile, results obtained by Ahadi (2006) in Iran demonstrated that psychological problems of these pregnant mothers are remarkably more, compared to women with normal pregnancies and even primiparous women (18). On the other hand, the painful experience of the previous pregnancy loss and its transmission to the current pregnancy cause some problems and concerns, which distinguish these pregnancies from others. Therefore, some interventions are required to in addition to controlling the sadness caused by the recent loss, mainly address the psychological aspects of the subsequent pregnancy following stillbirth, such as mood swings, emotional support, doubts and threats, and emotional blockage, by boosting mental energy, giving reassurance, and using conscious cognitive processing (7, 10, 11, 17).

Despite the progress made in improving prenatal care, the rate of stillbirth is globally high. Passion and tendency of mothers and sometimes the pressure from relatives for early pregnancy before culmination of sadness and complete elimination of mourning symptoms have rendered pregnancy after a pregnancy loss different and highly critical for mothers in terms of psychological and physical health, compared to normal pregnancies and even primiparous women. 

The majority of the previous studies have focused on sadness and mourning of mothers after stillbirth and paid little attention to the pregnancy subsequent to stillbirth. Meanwhile, pregnancy itself is a critical and stressful process, which could be intensified by a preceding stillbirth, highlighting the importance of intervention. Given the high vulnerability of these women and negative effects of anxiety on mental health of mothers, pregnancy outcomes, and even postnatal health, we aimed to determine the effect of psychoeducation on anxiety during subsequent pregnancy after stillbirth in women vising healthcare centers.

## Materials and methods

This two-arm, semi-experimental study with a pretest-posttest design was conducted among women with a history of stillbirth, who were pregnant again and visited healthcare centers affiliated to a university of medical sciences in southeast of Iran in 2017 to receive prenatal care services. The sample size was estimated at 45 cases based on the study by Akbarzadeh et al. (2013) with 95% confidence level and 90% test power and based on the following equation (19). Eventually, 100 individuals were evaluated in total (50 per group) to ensure accuracy of the results. 


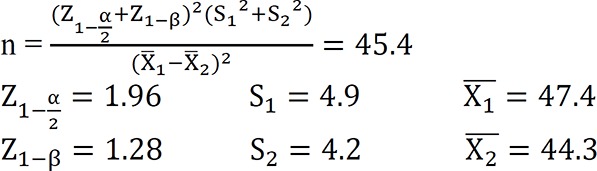


The inclusion criteria consisted of age above 18 years, less than 12 months interval between stillbirth and the subsequent pregnancy, natural pregnancy, and lack of high-risk pregnancy, singleton pregnancy, lack of experience of stillbirth or miscarriage more than once, gestational age of ≥ 20 weeks, lack of any recognized mental problems, no addiction, no psychosocial crisis, such as death of a relative, and no physical problems or serious diseases. The exclusion criteria included emergence of pregnancy complications (e.g., bleeding, leaking, and preeclampsia), hospitalization, pregnancy termination, and absence from more than one educational session. 

The data collection tool included a questionnaire containing two sections: 1) demographic characteristics and pregnancy-related information, such as age, occupation, level of education, gestational age, interval between stillbirth and the following pregnancy, and 2) Pregnancy Related Anxiety Questionnaire (PRAQ), which evaluates fears and concerns related to pregnancy. This questionnaire was designed by Wendenburg in 1989 (20). The short version of the mentioned questionnaire (PRAQ-17) contains 17 items. 

The exploratory factor analysis of the data in this questionnaire showed five factors of fear of delivery (three items), fear of bearing a child with physical or intellectual disabilities (four items), fear of change in marital relationship (four items), fear of change in moods and its impact on the child (three items), and self-centered fears or fear of changes in the mother's personal life (three items). The final score of the questionnaire is obtained by summing up all the item scores. Each item is scored based on a 7-point Likert scale. The total pregnancy anxiety score can be within the range of 17-119.

Psychometric properties of this questionnaire have been evaluated and confirmed by Karamouzian et al. (2016) in Iran. After confirming the face and content validity, the questionnaire was distributed among 170 pregnant women in Iran for psychometric assessment of the tool. The results of confirmatory factor analysis indicated an acceptable fit for the structure at the levels of the items and five subscales. Moreover, the significant correlation between components and the total scale of the questionnaire with Beck Anxiety Inventory established concurrent validity of the questionnaire. The test-retest coefficient for 40 pregnant mothers was within the range of 0.68-0.72 (p < 0.01), which confirmed the reliability of the inventory (21). In the present study, the reliability of the tool was estimated at the Cronbach’s alpha of 0.87 and within the range of 0.72-0.91 for the five factors.

After obtaining a formal letter of introduction from the Deputy of Research and Deputy of Health of the university, the researcher presented to the healthcare centers of the city. The necessary coordination was made with the relevant authorities prior to performing the research. At first, the eligible subjects were selected from the pregnant women with a history of stillbirth visiting the selected healthcare centers of the city to receive prenatal healthcare services in 2017. After receiving written informed consents, the subjects were randomly allocated to the intervention and control groups. At first, envelopes containing letters A and B for intervention and control, respectively, were prepared and randomly organized according to the total number of subjects. 

**Table 1:** Structure and educational contents of the psychoeducation sessions

**Session **	**Educational contents**
First	Introduction to unresolved sadness: a journey from the previous pregnancy and child to the present pregnancy and child, sympathy, and the effect of previous experience on the current pregnancy
Second	Psychological dimensions of pregnancy subsequent to stillbirth: threats, vulnerabilities, emotional blockage and shield, and expression of emotions
Third	Normal physiology of pregnancy, expressing the memories of pregnancy, and giving assurance
Fourth	Strategies to deal with pregnancy following stillbirth: stress management methods, problem solving models, and conclusion

Gradually and by identifying the eligible pregnant women, the envelopes were allocated to the selected participants in order. In addition, a pretest was taken from all the subjects in the intervention and control groups by filling out the short version of PRAQ, which contains 17 items. Personal details, telephone numbers, and address of the next appointment were obtained from the individuals assigned to the control group to collect posttest information at their home or in the clinic. 

The control group only received the routine prenatal care. In the intervention group, times of the educational classes were arranged with the subjects after obtaining their telephone numbers. The educational sessions were held in healthcare centers of the region according to the number of eligible pregnant women. The number of pregnant women attending each educational class varied from five to nine. Participants of the intervention group attended four psychoeducation sessions during four weeks and received the contents presented in table 1. After eight weeks, the research questionnaire was completed again by the participants at their home or in the clinics as the posttest. 

Contents of the educational package were collected from books, articles, and information resources related to the studies by O’Leary et al. (2004), Côté-Arsenault and Donato (2011), and Khanzadeh et al. (2017) (6, 10, 15). Moreover, the researcher asked the opinions of some specialists in Clinical Psychology, Counseling, Obstetrics, Gynecology, and Midwifery Education to make the necessary modifications. After applying the opinions of the experts, the final format of the content was designed by the research team. It is worth mentioning that the psychoeducation sessions were held by an individual with an MSc in Counseling in Midwifery and experience of working in maternity wards and health care centers under the supervision of a specialist. 

***Data analysis:*** Data analysis was performed in SPSS, version 21 using descriptive statistics (frequency, percentage, mean, standard deviation, and minimum and maximum), paired t-test (to compare the means of the study groups before and after the intervention), independent t-test (for inter-group comparison of means), and Chi-squared test (to compare the frequency of the qualitative variables of the intervention and control groups). P-values less than 0.05 were considered statistically significant. 

***Ethical considerations:*** This article was approved by the Ethics Committee of Zahedan University of Medical Sciences, Zahedan, Iran, with the code of IR.ZAUMS.REC.1369.59. At first, some information regarding the intervention process and time was provided to the participants and written informed consents were obtained prior to the study. In addition, the subjects were assured of the confidentiality terms regarding their personal data, and they were allowed to withdraw from the research at any time.

## Results

Shapiro-Wilk test results on the scores of pregnancy-related anxiety were indicative of normal distribution of the data. Therefore, the use of parametric tests in this study was approved. According to table 2, in which demographic characteristics of the participants are presented, mean age of the pregnant women was 27.90 ± 7.00 years in the intervention group and 58.58 ± 11.69 years in the control group. The majority of the women in the intervention (92%) and control (98%) groups were housewives. In addition, 82% and 80% of the women in the intervention and control groups had intentional pregnancy, respectively. In terms of educational level, only 20% of the intervention group and 12% of the control group had academic education.

The mean ages of pregnancy were 24.21 ±4.12 and 25.28 ± 3.83 weeks in the intervention and control groups, respectively. Moreover, the mean gravidities in the intervention and control groups were reported to be 4.00 ± 1.73 and 4.40 ±1.92, respectively. The mean intervals between stillbirth and the subsequent pregnancy were 7.06 ± 3.90 and 6.54 ± 3.82 months in the intervention and control groups, respectively. 

**Table 2:** Demographic Characteristics of intervention and control groups

**Variable**	**Intervention Group** **n (%)**	**Control Group** **n (%)**	**p value**
Desire for Pregnancy			
Yes	41(82)	40(80)	0.1
No	9(18)	10(20)	
Total	50(100)	50(100)	
Occupation			
Employee	4(8)	1(2)	0.36
Unemployed	46(92)	49(98)	
Total	50(100)	50(100)	
Education			
Illiterate	6(12)	12(24)	0.51
Lower than diploma	22(44)	21(42)	
Diploma	12(24)	11(22)	
Higher than diploma	10(20)	6(12)	
Total	50(100)	50(100)	
	**Mean ± SD**	**Mean ± SD**	
Age (Year)	27.90 ± 7.00	29.46 ± 7.78	0.29
Gestational age (Week)	24.21 ± 4.12	25.28 ± 3.83	0.84
Number of visits	4.44 ± 2.20	5.24 ± 1.67	0.04
Number of ultrasound Scans	2.62 ± 1.17	2.60 ± 0.98	0.91
Total Number of pregnancies	4.00 ± 1.73	4.40 ± 1.92	0.27
Interval of pregnancy after stillbirth (month)	7.06 ± 3.90	6.54 ± 3.82	0.5
Optimism to the progress of pregnancy	8.94 ± 1.30	7.83 ± 1.76	0.002

According to the independent t-test and Chi-squared test, no significant difference was observed between the study groups regarding the demographic characteristics (p > 0.05).

It was demonstrated that the mean numbers of physician and midwife visits were 4.44± 2.20 and 5.24 ± 1.67 in the intervention and control groups, respectively (p = 0.04). In addition, the mean numbers of ultrasound evaluations were 2.62 ± 1.17 and 2.60 ± 0.98 in the intervention and control groups, respectively (p = 0.91). The evaluation of optimism about the natural prenatal development on a scale of 0-10 showed that the mean levels of optimism in the intervention and control groups were 8.94 ± 1.30 and 7.83 ± 1.76, respectively. According to the results of the independent t-test, a significant difference was observed between the study groups in this regard (p = 0.002). 

According to table 3, the mean score of pregnancy-related anxiety in the intervention group decreased from 59.32 ± 21.12 to 50.64 ± 20.05 after the educational sessions. On the other hand, the mentioned value increased from 60.28 ± 23.75 to 63.54 ± 22.90 in the control group after the intervention. 

**Table 3:** Pregnancy anxiety scores in intervention and control groups before and after the psycho-education intervention

**Variable**	**Before** **Mean ± SD**	**After** **Mean ± SD**	**Changes** **Mean ± SD**	**p value**
Anxiety of pregnancy				
Intervention group	59.32 ± 21.12	50.64 ± 20.05	-8.68 ± 1.99	0.0001
Control group	60.28 ± 23.75	63.54 ± 22.90	+3.26 ± 7.12	0.002
Independent t test	0.83	0.003	0.0001	

Moreover, mean changes in the score of pregnancy anxiety in the intervention and control groups were reported to be -8.68 ± 1.99 and -3.26 ± 7.12, respectively. The independent t-test reflected a significant difference between the intervention and control groups after the educational sessions in terms of mean score of pregnancy-related anxiety (p = 0.003). Furthermore, a significant difference was found between the study groups regarding the mean changes in scores (p = 0.0001).

## Discussion

The core findings of this study, which was performed to determine the impact of psychoeducation on anxiety in subsequent pregnancy after stillbirth, reflected that the mean score of anxiety in the pregnant women with a history of stillbirth, who attended psychoeducation sessions, was significantly lower, compared to the control group. This was indicative of the effectiveness of the intervention in decreasing the level of pregnancy anxiety. Despite the large number of studies on prenatal education to increase psychological well-being of pregnant women, and emphasis of researchers on the unique and specific needs of pregnant women with a history of stillbirth and pregnancy loss, and recommendation of various interventional programs, there is a few number of interventions designed and tested on this vulnerable group women in developing countries, including Iran. 

In line with our findings, Tektas and Cam (2017) conducted a study on a sample of 101 pregnant women with a history of pregnancy loss. According to their results, provision of care according to Watson’ caring theory had a significant positive impact on the parameters of maternal mental health, including anxiety. In the mentioned study, the mean anxiety score of the intervention group (score = 5.7) decreased to half after the intervention, compared to the control group (score = 14) (22). The difference between the current study and the mentioned one was the use of Beck Anxiety Inventory, which evaluates general anxiety, and holding five sessions starting from weeks 10-12 through week 28 of pregnancy in the latter study. Meanwhile, PRAQ was applied in the current study and the intervention was carried out from the week 20 of pregnancy in the form of four sessions (one session per week), and anxiety level of the subjects was evaluated in the form of posttest near the time of delivery (week 32 of pregnancy). While the mean anxiety scores of the intervention and control groups were different after the educational sessions compared to the pretest, they had not decreased to a great extent, and the intervention was not only able to prevent increased anxiety, but also it led to a significant reduction in anxiety of the participants. Given the history of stillbirth in the subjects, it seems that their anxiety level increases approaching the time of delivery. These results could be justified by referring to the paired t-test results in Table 3, which indicates a significant difference in the mean anxiety score of the control group after the intervention. In contrast, in subsequent pregnancies following the loss of pregnancy, mothers’ hope for holding their baby in their arms is expected to increase as they approach delivery (23).

In order to determine the effectiveness of interventions designed to boost mental and psychological well-being of pregnant women following a pregnancy loss, Carrera et al. (1998) marked that the depression level of pregnant women with a history of miscarriage, who received social support interventions for one year, was significantly lower, compared to those not receiving any social support. In the mentioned study, even the depression level of the subjects in the intervention group was somewhat similar to those who gave birth to healthy children in their previous pregnancies (24). A qualitative research was conducted by Meredith et al. (2017) to assess the experience of women about the establishment of clinics for subsequent pregnancies following miscarriage as specialized healthcare centers for this group of women in Australia. According to their results, visiting these centers diminished anxiety and increased emotional support and receiving midwifery care (13).

Despite the limited number of interventional studies in this area, there have been some studies not showing any specific effect on improved mental condition and decreased anxiety and stress of mothers with a history of stillbirth or miscarriage. Contrary to our findings, the study by Cote-Arsenault et al. (2014) demonstrated that the provision of home-based care had no significant impact on reduced levels of anxiety and depression and enhanced maternal-fetal bond in pregnant women after an experience of stillbirth (23). Nevertheless, the subjects were greatly content with social support during this period. This discrepancy in results might be due to the time of posttest (at week 34), limited sample size (n = 24), cultural differences, or content and type of intervention. One of the coping strategies required by pregnant women after miscarriage is social support (13). Another cause of effectiveness of the psychoeducation sessions in the current study might be the application of group intervention, compared to the use of individual intervention in the study by Cote-Arsenault et al. (2014).

In comparison of the current results with those of other studies, it must be considered that women with a history of stillbirth (loss of child after 22 weeks of pregnancy) participated in the present study, while the subjects of the similar studies were women with a history of miscarriage (even before week 22 of pregnancy), which distinguishes the effectiveness and type of interventions. In this regard, some believe that age of pregnancy in the previous loss could not predict loss and anxiety in the current pregnancy. However, women who lost their child at a higher age of pregnancy reported increased anxiety level as pregnancy progresses and approaches the time of the previous loss (11). 

Wright (2005) and Cote-Arsenault and Feije (2004) believed that women with a history of previous miscarriage need communication and support. Communication skills, especially active listening, are significantly crucial for the treatment of these women. Effective and therapeutic relationships facilitate understanding the needs of these women, which when coupled with the provision of information about pregnancy and relaxation practices that are practical intervention can decrease their anxiety level (25, 26). In the current study, one psychoeducation session was allocated to learning stress management skills, such as deep breathing, progressive muscle relaxation, and aromatherapy. It seems that application of these techniques, especially deep breathing, which is significantly helpful at the onset of labor pain, could lead to decreased anxiety level.

Women with a history of pregnancy loss frequently feel that their experience is considered as a minor issue by friends, families, and even healthcare providers and that their fears and anxiety are neglected (23, 25). In this study, the participants were satisfied with the free discussion of their experience instead of using an emotional shield and reactive defense mechanisms, avoiding, and rationalizing, which created a sense of sympathy and decreased the level of anxiety. 

It seems that the intervention designed in the present study encouraged women to express their memories of the previous pregnancy, emotions, and positive and negative thoughts, use conscious rumination, and accept emotions from the instructor and other group members. This encouragement for asking questions enables women to have a positive feeling about their pregnancy. In this respect, Côté-Arsenault (2007) marked that talking about contradictory emotions (fear and hope) with healthcare providers in a safe and judgment-free environment is significantly essential for the regulation of emotions and creation of positive feelings, especially during pregnancy (11). Pregnancy after a loss can be normalized by listening to other women with similar emotions and confirming the anxiety caused by pregnancy by members and instructors, hence decreased anxiety level in these individuals. 

Another coping strategy in this regard is acquisition of knowledge and information. In a qualitative study by Meridith et al. (2017), it was found that issues could be dealt with in a more efficient manner if there is sufficient information (13). Some parts of the psychoeducation sessions in that study were review of subsequent pregnancy following stillbirth and its difference with normal pregnancy, providing information on unresolved sadness caused by pregnancy loss and the growth and development of infants in a safe environment. It seems that this information could alleviate anxiety and hopelessness. In line with our findings, women who had information about pregnancy subsequent to stillbirth were significantly more optimistic about the progress and health of their pregnancy, compared to the control group. In this regard, Smith and Smart (2014) published a book entitled as “An Unforgettable Love” for pregnant women with a history of miscarriage, providing information for women of the target group. According to their results, the information of the book significantly decreased women’s distresses (27).

To confirm the effectiveness of the psychoeducation intervention in the current study, it should be mentioned that emotional tools, reassuring, and decreased anxiety of the intervention group significantly decreased the number of unnecessary visits to the clinics. However, this intervention had no effect on the number of ultrasound evaluations, which might be because the number of ultrasounds is not generally high in pregnant women in our society. In this regard, the mean number of ultrasound was two in the intervention and control groups. In the study by Côté-Arsenault and Earl and Donato (2006), the number of healthcare provider visits of women with subsequent pregnancy after stillbirth or miscarriage was significantly higher as they want to be constantly assured of the fetal health and undergo a more extensive series of examinations (28).

***Limitations of the study:*** The relatively large sample size, use of a multidimensional intervention (emotional, supportive, educational, and collective), and implementation of the intervention by a midwife with counseling specialty are some of the strengths of the current study. Subjects of the control group were somewhat dissatisfied due to the lack of receiving any interventions. However, the thought of making progress in this area, which leads to helping women in similar situations, appeased them. While the anxiety assessment tool we used was slightly more specialized (PRAQ), compared to those employed in similar studies, lack of presence of a specific scale for the evaluation of pregnancy anxiety following a previous loss was one of the limitations of the current and similar studies. Use of psychoeducation intervention throughout pregnancy would be more efficient to accurately determine the effect of constant care using this approach. Since the importance of child bearing and reactions to pregnancy loss are affected by personality, ethnic, religious, cultural, and social factors, the results must be generalized with caution. Future studies are recommended to evaluate the effect of psychological intervention on other relevant variables, such as hope and maternal-fetal bond, and the effect of outcomes on fathers.

## Conclusion

Currently, no proper education or intervention based on the specific needs of pregnant women with a history of stillbirth is provided in healthcare centers. There is no opportunity for effective communication and providing a supportive environment to encourage women express their emotions. In this respect, the results of the current study indicate the significance of design and positive and significant effect of specialized interventions for women with subsequent pregnancy following stillbirth. Therefore, we recommend taking into account the effect of previous pregnancy loss on the current pregnancy by healthcare providers through providing psychoeducation interventions in conjunction with the routine care for pregnant women. In addition, our findings led to the provision of a primary model for designing and responding to psychological needs of pregnant women with a previous pregnancy loss. Meeting the emotional and information needs of these women enables them to protect themselves and their child not only during pregnancy but also after it.
